#  Time to regain lost ground: Tuberculosis in the COVID-19 era

**DOI:** 10.2807/1560-7917.ES.2021.26.24.2100564

**Published:** 2021-06-17

**Authors:** Dominik Zenner

**Affiliations:** 1 Centre for Global Public Health, Institute for Population Health Sciences, Queen Mary University London, United Kingdom

**Keywords:** COVID-19, Tuberculosis control, TB reference laboratories

For 18 months, the coronavirus disease (COVID-19) pandemic, caused by the rapid global spread of severe acute respiratory syndrome coronavirus 2 (SARS-CoV-2), has led to well over 3.5 million recorded deaths worldwide [1] in addition to substantial short and long-term morbidity. Its highly efficient transmission, mostly via the respiratory route, and the consequently high reproduction number has made epidemic control a formidable challenge, with widespread impact on health systems and societies. Comprehensive social and public health measures including movement restrictions have been implemented in most European countries. Often accompanied by restructuring and reprioritisations in healthcare systems, these measures have also had a documented impact on a wide range of non-COVID-19-related aspects of health, including non-communicable diseases or maternal and child health [2,3]. Reasonable and justifiable emergency measures may initially be necessary to curb the pandemic. However, it is also important to uphold services for other infectious and non-infectious diseases in order to maintain achievements and to continue progress to “*significantly improve the health and well-being of populations”* [4].

Faced with the urgency for pandemic control, it may be challenging to keep the morbidity and mortality of other diseases in mind. Globally, tuberculosis (TB) was the deadliest infectious disease before the COVID-19 pandemic, with ca 10 million cases and around 1.5 million deaths worldwide each year [5,6]. Initial modelling studies predict that COVID-19 and associated measures will likely lead to significant increases in TB incidence and mortality over the next few years [7,8]. The World Health Organization (WHO) predicts that even a 50% reduced TB notification rate over a 3-month period may translate into 400,000 additional cases alone [6]. While the extent of TB service disruption may depend on the context in each country, such as health service capacity, barriers to care or degree of movement restrictions, early observational data seem to corroborate the substantial impact on TB services at multiple levels [9]. Acknowledging this, the WHO, alongside others, has repeatedly emphasised the need to refocus on TB control [10]. Similar to TB, there is mounting evidence that COVID-19 may also aggravate health inequalities, that social determinants are comparable [11] and that their co-occurrence is linked to worse outcomes [12], supporting the notion of a syndemic [13]. Granular observational data, which describe changes in TB incidence, mortality and disease in different population groups and the incidence and outcomes of TB-COVID-19 coinfections, are urgently needed to improve understanding of the impact on TB control and identify lessons learned.

In this issue, Maurer et al. report on a survey of National TB Reference Laboratories in the WHO European Region, documenting the impact of COVID-19 on TB laboratory services, including countries with high TB multidrug resistance [14]. The authors found that many countries experienced a significant increase in workload as a result of COVID-19, without much additional staff and resources, and saw a concomitant reduction of TB laboratory work. These findings corroborate those in an earlier survey, which covered the European Union/European Economic Association countries and the UK, and focused on laboratory impact within the European Reference Laboratory Network [15].

Dara et al. analyse the public health aspects of TB care during the COVID-19 pandemic, also in this issue [16]. Based on results from a survey on European national TB control programmes in the WHO European Region, they report significant decreases of TB notifications and estimated a potentially substantial impact on TB transmission and fatalities caused by diagnostic delay [16]. The reasons for these observations are likely complex and may include direct effects of the pandemic by stretching already weak health systems, for example, or indirect impact through healthcare access barriers or societal and movement restrictions.

With successful COVID-19 vaccination programmes now well underway in many countries, it will be important to conduct further scientific analyses to gain reliable estimates of the benefits, harms and costs of the various COVID-19 pandemic measures. These estimates need to be considered alongside the impact of the disease itself as well as in the context of other diseases and wider aspects of health and wellbeing to inform public health decision-making. Now is also the right time to renew our commitment to ‘end TB’ [17] and strengthen all aspects of TB control, including diagnosis, treatment and preventive therapy in keeping with commitments made at the first United Nations High-Level Meeting on TB in 2018 [18]. This process may take considerable amount of time, effort and resources. However, it will be well worth it to ensure we can regain lost ground vis-à-vis the End TB strategy [17], make similar progress in other disease areas and learn valuable lessons to be better prepared for the next public health emergency.
